# Perception of quality of care using patient reported experience measures (PREMs) in a cohort of adults with HIV: A cross-sectional study

**DOI:** 10.1097/MD.0000000000033442

**Published:** 2022-04-07

**Authors:** Elisa de Lazzari, Leire Berrocal, Emma Fernández, Montserrat Laguno, Iván Chivite, Berta Torres, Ana González-Cordón, Lorena de la Mora, Juan Ambrosioni, Alexy Iniciarte, José Luís Blanco, Josep Maria Miró, Esteban Martínez, Maria Martínez-Rebollar, Josep Mallolas

**Affiliations:** a CIBERINFEC, Instituto de Salud Carlos III, Madrid, Spain; b HIV Unit. Hospital Clínic-IDIBAPS. University of Barcelona, Barcelona, Spain.

**Keywords:** HIV, HRQoL, PREMS

## Abstract

Human immunodeficiency virus (HIV) infection is considered a chronic disease. Antiretroviral therapy has allowed persons with HIV (PLWHIV) to achieve the 90-90-90 objectives proposed by the World Health Organization for 2020; but an additional challenge is getting an adequate health-related quality of life. A determining factor in the health-related quality of life of PLWHIV is the health care they perceive to receive. In this sense, we aimed to assess the perception of the outpatient care provided and to identify possible areas for improvement in a single-center, cross-sectional study at the HIV unit of Hospital Clínic, Barcelona. We sought patient reported experience measures by an anonymous e-survey with 11 statements based on a 1 to 6 Likert scale, and a final question measuring user satisfaction and loyalty through the Net Promoter Score (NPS). All PLWHIV with at least a clinical visit between January 1, 2020 and October 14, 2021 were invited. Of 5493 PLWHIV e-mailed, 1633 (30%) responded to the survey. The overall evaluation of clinical care was very favorable. The evaluation of the physical environment and facilities and the time spent in the waiting room received the lowest scores. According to the Net Promoter Score test results, 66% of respondents were willing to recommend this service, and 11% were not. Thus, monitoring patient reported experience measures in PLWHIV actively receiving outpatient care in our hospital allowed to identify the users’ perception on quality of the care received, to determine the rate of satisfaction with the care, and to identify areas for improvement.

## 1. Introduction

“Health-related quality of life”^[1]^ (HRQoL) is defined as the perception that a patient has regarding the effects of a disease or the application of a certain treatment in areas of his or her life, especially on his or her physical, emotional and social well-being.

In the last century, the substantial development of new pharmacological products and health technology has increased the interest on the measurement of quality of life in health care.

Traditional medical outcome variables such as clinical symptoms, survival rate, and safety and clinical efficacy of a drug are no longer sufficient to adequately reflect the effect of health care and interventions, and it is considered necessary to analyze the perspective of the person with a disease too. What matters is how the person with a disease feels rather than how health professionals believe they should feel based on clinical measures.^[2]^ In that way, whether therapy can achieve a life worthy of being lived, both from the social and psychological perspective as well as the physical perspective, should be assessed.

HRQoL has become an important measure of the impact of medical care. Currently, the focus is on the quality, rather than the quantity, of life. Gradually, the patient experience has been incorporated into analyses of care processes to evaluate quality from the perspective of the person receiving the service^[3]^ to improve it. For this, new assessment tools have been introduced: PROMs (*patient-reported outcome measures*), related to treatment objectives, and PREMs (*patient-reported experience measures*), related to the experience of a treatment or intervention.^[4–6]^

The management of human immunodeficiency virus (HIV) infection has been changing over the last decades, adapting to the needs of persons living with HIV (PLWHIV) and, above all, influenced by the available therapeutic arsenal. Currently, the high efficacy of antiretrovirals, with the consequent sustained viral suppression and disappearance of opportunistic diseases, has drastically reduced the mortality associated with HIV^[7–9]^ in way such that PLWHIV show a life expectancy similar to that of the general population, turning HIV infection into a chronic disease.^[10]^

In addition to the goals outlined by the World Health Organization to control HIV infection for 2020,^[11]^ that is, 90% of PLWHIV diagnosed, 90% treated and 90% have undetectable viral load, a fourth objective focused to achieve a correct quality of life in 90% of these patients has been also raised in recent years.^[12]^

The current challenge of the outpatient units caring for PLWHIV is to redefine what the optimal model of care should be, how to adapt resources according to the needs of each patient and how to incorporate this fourth 90% goal related to quality of life.

In recent years, tools have been introduced for the collection of patient-reported outcome measures in PLWHIV within clinical trials and controlled studies^[13–17]^ to assess the possible benefits and risks of an intervention more accurately; however, the information reported in the literature on PREMs in PLWHIV is scarce.^[18,19]^

In the HIV Unit of the Hospital Clinic of Barcelona, nearly 6000 PLWHIV are currently receiving outpatient care, and health workers at the Unit have been committed for years to updating and improving the approach and care of them. In addition to assess the quality of care with well-established indicators of safety and efficacy,^[20]^ we aimed to know about the experience of PLWHIV, which some authors consider the third pillar in the evaluation of quality of care and a key element for improving health care in the context of person-centered care.^[21]^

The objective of this study was to assess the user perception on quality of the care received, to determine the rate of satisfaction with the care and to identify potential areas for improvement.

## 2. Methods

This was a cross-sectional study that evaluated through an anonymous electronic survey (e-survey) the perceived satisfaction of PLWHIV with the care received in the HIV Unit of the Hospital Clinic of Barcelona. All patients for whom we had records for at least 1 visit between January 1, 2020 and October 14, 2021 were considered candidates to respond the survey. Two analysis instruments were used, a PREM assessment survey and the net promoter score (NPS).

### 2.1. Instruments

The survey was prepared with the help of the Patient Care Research Team at our hospital, taking as a model similar surveys carried out in other areas of care for chronic patients of the Hospital Clinic of Barcelona and the recommendations of the Agency for Quality and Health Assessment of Catalonia.^[22]^ The questionnaire includes 5 questions regarding demographic information to determine the profile of the respondent and 11 statements scored by the respondent based on the degree of disagreement or agreement using a Likert scale ranging from 1 to 6. These statements are as follows:

Q1 The location where the consultation occurred was appropriate.
Q2 The time spent in the waiting room was acceptable.
Q3 The information received from the professionals who treated me was clear and concise.
Q4 The vocabulary used was adequate.
Q5 The information received during the visit responded to my needs.
Q6 I believe my opinion mattered, and I felt like a participant in decision-making about my health.
Q7 The visit was carried out in a climate of trust that allowed me to express my concerns.
Q8 The time allocated to doubts and/or questions was sufficient.
Q9 The total length of the consultation seemed to be the right amount.
Q10 I feel that the attention received during the visit was adequate.
Q11 The time spent in the consultation was worth it (considering travel, waiting time, etc).

In addition to the survey, a final question, “How likely is that you would recommend this Service to a friend or colleague?,” based on a 0 to 10 scale was associated with measuring user satisfaction and loyalty with our service through the NPS indicator.^[23]^

The electronic questionnaire was implemented in 3 languages: English, Spanish and Catalan, using the online survey tool LimeSurvey hosted at Hospital Clinic of Barcelona.

The system was programmed to mass-send the survey to the registered email of all PLWHIV for whom there were clinical records for at least 1 visit between the stablished period, January 1, 2020 and October 14, 2021. Seven and twelve days after the initial email on 10/14/2021, the system automatically sent 2 reminder emails to PLWHIV who had not yet responded.

### 2.2. Statistical analysis

Subjects were classified into 3 groups based on the scores obtained from the NPS question: those scoring 9 or 10 are considered *Promoters* (they would recommend this service to a friend or family member); those scoring 7 to 8, *Passives*, and those scoring 0 to 6 *Detractors*. The final NPS score was calculated as the difference between the percentage of *Promoters* and *Detractors*. Qualitative characteristics were described using absolute frequencies and percentages and were compared among the NPS groups with the chi-square test. Quantitative variables were summarized as the mean and standard deviation (SD), and comparisons among the NPS groups were conducted using analysis of variance. The pattern of relationships among the responses to the questionnaire was analyzed using the multiple/joint correspondence analysis (MCA) with 2 dimensions to capture the Guttman effect that affects preference data and occurs when higher order dimensions are polynomial functions of the first one. In our data the arch shaped form suggests a non-symmetric quadratic function. In the MCA graph the first dimension defines a scale of scores from worst to best, from left to right, and the second defines a contrast between extreme responses (positive part of the axis) and neutral/moderate responses (negative part of the axis). The NPS groups were used as a supplementary variable. All tests were 2-tailed with a 95% confidence level. Statistical analyses were performed with Stata (StataCorp. 2021. *Stata Statistical Software: Release 17*. College Station, TX: StataCorp LLC).

## 3. Results

**Figure 1.**
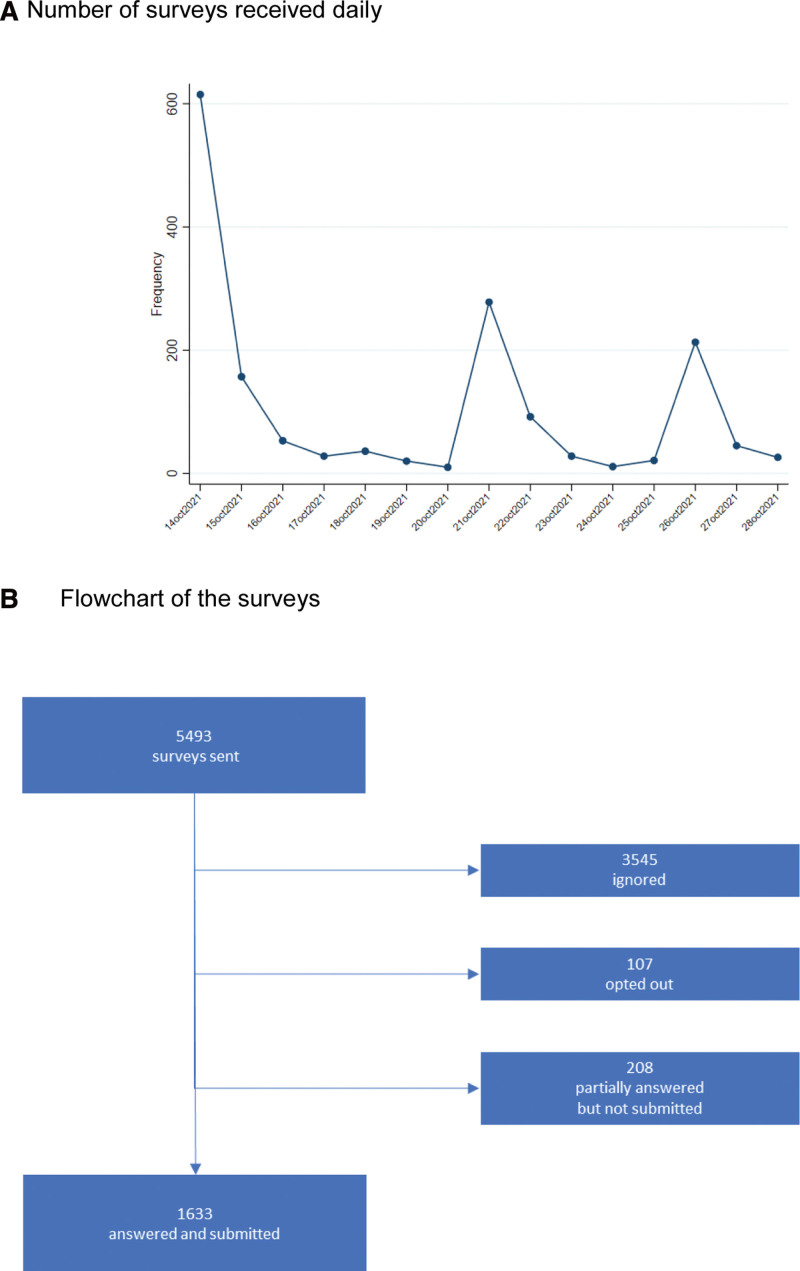
Surveys: (A) Number of surveys received daily. (B) Flowchart of the surveys.

The e-survey was submitted to 5493 PLWHIV. Figure 1A shows the number of surveys received daily. We collected 1633 (30%) correctly completed surveys, and 208 (4%) respondents only partially completed the questionnaire. One hundred and 7 (2%) PLWHIV accessed the e-survey invitation but did not participate. Figure 1B shows the flowchart of the surveys. Sixty-eight percent (n = 1250) of the surveys were answered in Catalan, 30% (n = 559) in Spanish and 2% in English (n = 32).

Most PLWHIV who responded were older than 35 years (90%) and the respondents were evenly distributed among the 3 age groups outlined in the questionnaire. Fifty-eight percent had received higher education, and only 8% reported primary education as their highest level. Two-thirds of the respondents were of Spanish origin, 10% were from other European countries and 21% were from Latin America. Sixty percent had a long-standing HIV infection diagnosed more than a decade ago. Baseline characteristics of the population are shown in Table 1. The age distribution for the cohort of responders was roughly similar to that of the whole cohort; the percentage older than 45 years was 63% and 54%, respectively. The percentage of PLWHIV with higher education (58% vs 46%) and of Spanish origin (66% vs 53%) was higher in the cohort of responders than in the whole cohort.

**Table 1:** Characteristics of the population who responded to the survey.

Variable	Summary statistics
Age*	
Under 35 yr	164 (10%)
36–45 yr	441 (27%)
46–55 yr	513 (31%)
Over 55 yr	513 (31%)
*Total*	*1631 (100%*)
Education level*	
Primary	134 (8%)
Secondary	551 (34%)
University	943 (58%)
*Total*	*1628 (100%*)
Origin*	
Spain	1074 (66%)
Europe	155 (10%)
Latin America	348 (21%)
North Africa	1 (0%)
Sub-Saharan Africa	6 (0%)
Asia	10 (1%)
USA/Canada	16 (1%)
Other	18 (1%)
*Total*	*1626 (100%*)
Year of diagnosis*	
1980–1989	104 (7%)
1990–1999	327 (22%)
2000–2009	470 (31%)
2010–2021	600 (40%)
*Total*	*1501 (100%*)
Residence*	
Barcelona	1397 (94%)
Girona	22 (1%)
Tarragona	40 (3%)
Lleida	13 (1%)
Rest of Spain	14 (1%)
*Total*	*1484 (100%*)

* n (column percentage).

**Figure 2.**
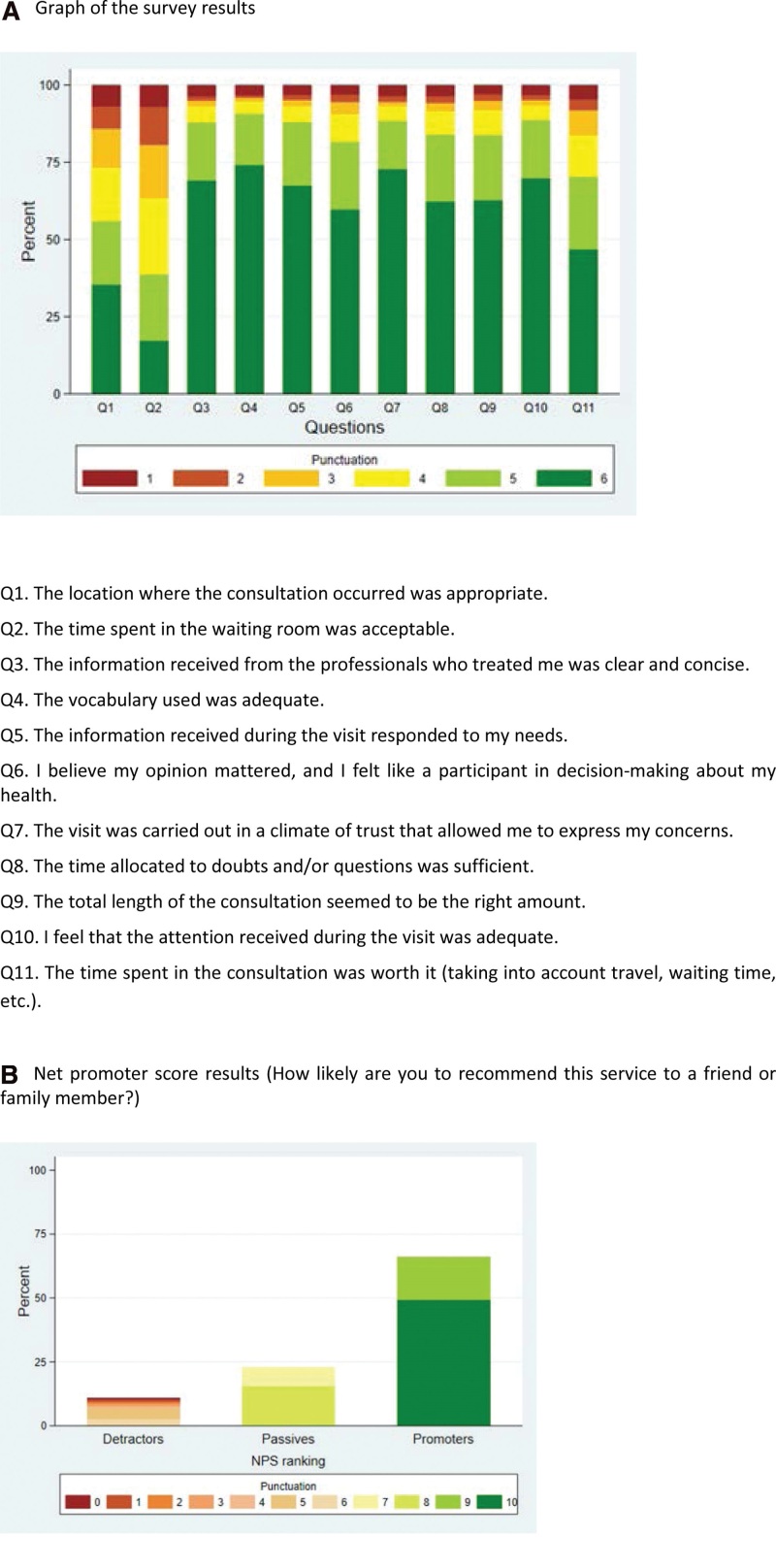
Results: (A) Graph of the survey results. (B) Net promoter score results (How likely are you to recommend this service to a friend or family member?).

Overall, the average score for all the statements in the survey was above 4; that is, the responses were favorable, except that for question Q2 related to the time spent in the waiting room that was the worst-rated statement with an average score of 3.9 (SD 1.5) and viewed unfavorably (≤3) by 36% of the users (see Fig. 2A and Table 2). Question Q1, which assesses the physical environment and facilities, received an average score of 4.4 (SD 1.6), and 27% of users felt that the room for consultation was inadequate. Questions Q3, Q4, and Q5, which address how medical information is delivered and received, were very well rated, with average scores ≥ 5.4 (SD 1.2). The clarity of the information received from health care professionals as well as the language used were considered adequate (≥4) for more than 93% of respondents; in fact, Q4 was the highest scoring statement among those in the questionnaire. Finally, 93% of the respondents indicated that the information received responded to their needs. Question Q6, analyzes how the user perceived their involvement in decision making and 91% of the participants indicated that it was adequate. The following group of questions, from Q7 to Q10, delve into the assessment of the quality of the visit: Q7 assesses the development of a climate of trust in which concerns can be expressed, Q8 and Q9 assess time allocated for questions and total visit length and Q10 assess attention during the visit. All the responses in this area were very positive, with 90% of respondents agreeing with the survey statement. For the last question, Q11, which assesses overall experience, the average score was 4.9 (SD 1.4) and 84% of the users responded that the time spent in the consultation was worth it, taking into considering travel, time and waiting.

**Table 2:** Survey.

	Summary statistics	
Variable	n (Column percentage)	Arithmetic Mean (SD) [n]
The location where the consultation occurred was appropriate*		
1	113 (7%)	4.44 (1.57) [1629]
2	117 (7%)	
3	207 (13%)	
4	279 (17%)	
5	336 (21%)	
6	577 (35%)	
*Total*	*1629 (100%*)	
The time spent in the waiting room was acceptable*		
1	115 (7%)	3.93 (1.48) [1629]
2	201 (12%)	
3	283 (17%)	
4	398 (24%)	
5	351 (22%)	
6	281 (17%)	
*Total*	*1629 (100%*)	
The information received from the professionals who treated me was clear and concise*		
1	61 (4%)	5.41 (1.17) [1630]
2	21 (1%)	
3	31 (2%)	
4	83 (5%)	
5	308 (19%)	
6	1126 (69%)	
*Total*	*1630 (100%*)	
The vocabulary used was adequate*		
1	55 (3%)	5.52 (1.08) [1630]
2	11 (1%)	
3	23 (1%)	
4	63 (4%)	
5	270 (17%)	
6	1208 (74%)	
*Total*	*1630 (100%*)	
The information received during the visit responded to my needs*		
1	53 (3%)	5.41 (1.14) [1630]
2	26 (2%)	
3	33 (2%)	
4	82 (5%)	
5	336 (21%)	
6	1100 (67%)	
*Total*	*1630 (100%*)	
I believe my opinion mattered, and I felt like a participant in decision-making*		
1	51 (3%)	5.23 (1.23) [1627]
2	39 (2%)	
3	65 (4%)	
4	143 (9%)	
5	356 (22%)	
6	973 (60%)	
*Total*	*1627 (100%*)	
The visit was carried out in a climate of trust that allowed me to express my concerns*		
1	59 (4%)	5.45 (1.17) [1630]
2	30 (2%)	
3	23 (1%)	
4	75 (5%)	
5	257 (16%)	
6	1186 (73%)	
*Total*	*1630 (100%*)	
The time allocated to doubts and/or questions was sufficient*		
1	59 (4%)	5.28 (1.23) [1628]
2	35 (2%)	
3	46 (3%)	
4	120 (7%)	
5	354 (22%)	
6	1014 (62%)	
*Total*	*1628 (100%*)	
The total length of the consultation seemed to be the right amount*		
1	49 (3%)	5.30 (1.19) [1631]
2	34 (2%)	
3	51 (3%)	
4	129 (8%)	
5	344 (21%)	
6	1024 (63%)	
*Total*	*1631 (100%*)	
I feel that the attention received during the visit was adequate*		
1	53 (3%)	5.44 (1.14) [1629]
2	26 (2%)	
3	30 (2%)	
4	75 (5%)	
5	308 (19%)	
6	1137 (70%)	
*Total*	*1629 (100%*)	
The time spent in the consultation was worth it (taking into account travel, waiting time, etc)*		
1	76 (5%)	4.88 (1.41) [1620]
2	56 (3%)	
3	134 (8%)	
4	214 (13%)	
5	382 (24%)	
6	758 (47%)	
*Total*	*1620 (100%*)	

* n (column percentage).

The average NPS was 8.7 (SD 1.9). Sixty-six percent of the users were classified as promoters. Only 11% scored below 7 and, therefore, were classified as detractors. Thus, the net value of the resulting NPS was 55 (see Fig. 2B). For the NPS results, we did not assess differences by age group or education level, but clearly, Spanish respondents were less likely than respondents of any other origin to be classified as promoters (*P* = .0005).

**Figure 3.**
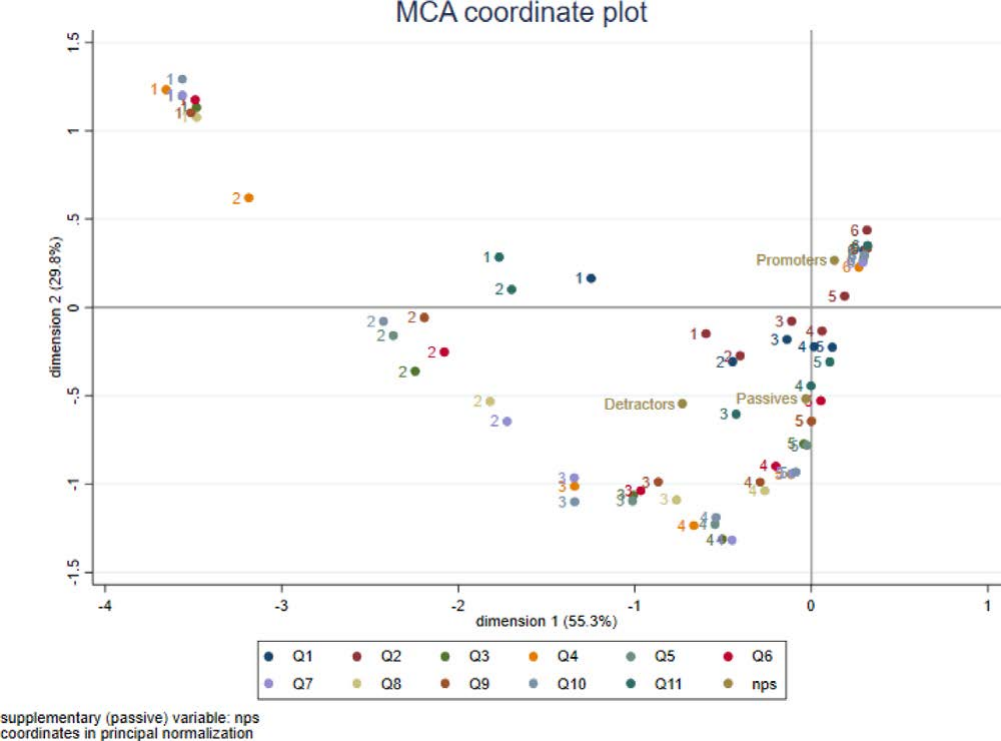
Multiple correspondence analysis.

In the graph of the MCA (Fig. 3), respondents who were considered promoters were associated with the maximum score, 6, for most questions, given their closeness to these scores. Respondents who were considered passives differed slightly from the previous group, being closer to scores of 4 and 5. The detractors, are further from the center, in the negative part of the axis, because they represented the group who provided the lowest scores.

The groupings or clusters of scores indicated that there was a pattern in these responses: those who responded with a 1 to 1 of the statements tended to do the same to other statements (except for statements about the location of the consultation (Q1), waiting time (Q2) and time spent in total (Q11)), as indicated by the cluster at the top left. The same occurred with the cluster for the score of 6, for which there was a grouping for all statements. The other scores were more dispersed, indicating more variation in the responses.

## 4. Discussion

The availability and efficacy of antiretroviral treatment have allowed to achieve the goals proposed by the World Health Organization for controlling HIV infection by 2020^[11]^ in our cohort. Specifically, of the 5493 PLWHIV followed in the HIV Unit of the Clinic Hospital of Barcelona, 99% were receiving antiretroviral treatment, and 94% had an undetectable viral load during the study period.

In accordance with the redefinition of therapeutic success proposed by the scientific community,^[12]^ which includes quality of life as a fourth goal to be achieved in 90% of PLHIV, we analyzed the quality of care we provide in our unit by reviewing the model of care offered to users and incorporating patient perception through an e-survey, with the ultimate goal of improving the service.

First, the response rate to the online invitation to participate in the study was remarkably high considering that PLWHIV had not been previously informed, nor the project was publicly advertised. One-third of the candidates answered our request, a percentage very similar to that obtained in an electronic format survey conducted in a different therapeutic area of the hospital.^[24]^ These data suggest that PLWHIV are sensitive to collaborating in a project that can help improve their care.

The demographic profile of the people who responded to the survey differed slightly from the average profile of PLWHIV of the unit: higher percentage of people of Spanish origin and higher education among the survey respondents. These subtle differences could indicate a selection bias about the persons included in the study. The digital divide existing in the population served in our unit may explain why some users did not feel comfortable with the electronic format and highlights the need to combine different strategies when presenting the survey that allows us to obtain responses from a greater number of users.

In general, we highlight the high percentage of positive responses to most of statements, with average scores >5, especially for those statements that evaluate the handling of information during the consultation and the perception of the quality of the visit. Thus, the overall assessment of the care received was 5.4 points out of 6. However, we must emphasize that there are issues related to space and time management worst rated and we must view this information as an opportunity to improve our service.

The waiting time prior to the consultation was reported as inadequate for almost half of the users, thus the corresponding statement was the lowest scored on the survey.

Similarly, 1-third of the users felt that the environment in which the consultation was carried out was inadequate. This result indicates the need to remodel this space, taking into consideration the opinion of the users to cocreate friendly environments where they feel comfortable and are guaranteed to have a good experience. Nine percent (9%) of respondents expressed disagreement with the role they have in decision-making. Although this is a small percentage, these data highlight the need to continue working on a patient-centered model where patients they perceive that their opinions are heard. There are few published data on satisfaction reported by PLHIV regarding the care they receive^[18,19]^ in general, satisfaction is favorable, similar to our data, but the results are difficult to compare given that different PREMs were used.

In addition to the survey, we have used the NPS, a simple and validated tool that allows a quick and simple analysis of whether things are being done well in a job or service. Although this tool was developed 2 decades ago,^[23]^ its use in the health field has been more recent and focuses on the benchmarking of private services. To date, there are no data in the literature on its use in the care of PLHIV. A NPS value above 50, such as that obtained in our study, is considered a high value and is synonymous with a positive score. Values similar to those obtained in this study, a NPS of 55, have been reported in studies conducted in the private health sector^[25,26]^ of our setting. We are struck by the fact that Spanish respondents were more critical in their assessment and, therefore, significantly lowered the percentage of respondents classified as promoters. On the contrary, the responses of non-Spanish origin were more favorable.

We are aware that simply collecting data regarding the experience of patients is not sufficient, and that it is necessary to take action, as recommended by experts through PREMs.^[27]^ The purpose of this study was to identify areas that need improvement, and we will evaluate NPS annually to verify whether any improvements impact the HRQoL perceived by the users.

This study has some limitations. Firstly, a bias for self-selection may have affected our results since only 1-third of the patients responded and their socio-demographic profile differed slightly from the average profile of the entire PLWHIV cohort who attended our hospital, as previously mentioned. Secondly, we don’t have prior experience in large e-surveys sent to our patients with which compare the response rate. Finally, there is little published data on PREMS in PLWHIV to compare our findings to, and there is no data regarding NPS in the specific HIV infection field. We see this study as a starting point for further research in this area.

In conclusion, monitoring patient reported experience measures in PLWHIV actively receiving outpatient care in our hospital allowed to identify the users’ perception on quality of the care received, to determine the rate of satisfaction with the care and to identify areas for improvement. The incorporation of the PREMs in the HIV units is essential to continue working on the improvement of the HRQoL of our patients. The periodic review of these tests will evaluate the effectiveness of the changes implemented after the needs detected have been addressed.

## Acknowledgments

We would like to thank Dr Escarrabill of the Patient Experience Evaluation Team of the Hospital Clínic for his invaluable help in the preparation of the survey and his advice on the use of PREMs for patients with HIV chronic infection.

## Author contributions

**Conceptualization:** Elisa de Lazzari, Leire Berrocal, Emma Fernandez, Montserrat Laguno, Iván Chivite, Berta Torres, Ana González-Cordón, Lorena de la Mora, José Luís Blanco, Josep Maria Miró, Esteban Martínez, Maria Martínez-Rebollar, Josep Mallolas.

**Data curation:** Elisa de Lazzari, Leire Berrocal, Emma Fernandez, Montserrat Laguno.

**Formal analysis:** Elisa de Lazzari, Leire Berrocal, Emma Fernandez, Montserrat Laguno.

**Funding acquisition:** Montserrat Laguno, Maria Martínez-Rebollar.

**Investigation:** Montserrat Laguno, Iván Chivite, Berta Torres, Ana González-Cordón, Lorena de la Mora, José Luís Blanco, Josep Maria Miró, Esteban Martínez, Maria Martínez-Rebollar, Josep Mallolas.

**Methodology:** Elisa de Lazzari, Leire Berrocal, Emma Fernandez, Montserrat Laguno.

**Validation:** Elisa de Lazzari, Leire Berrocal, Montserrat Laguno, Iván Chivite, Berta Torres, Lorena de la Mora, Juan Ambrosioni, Alexy Iniciarte, José Luís Blanco, Josep Maria Miró, Esteban Martínez, Josep Mallolas.

**Supervision:** Montserrat Laguno, Esteban Martínez, Maria Martínez-Rebollar, Josep Mallolas.

**Writing – original draft:** Elisa de Lazzari, Montserrat Laguno, Josep Mallolas.

**Writing – review & editing:** Montserrat Laguno, Esteban Martínez, Maria Martínez-Rebollar, Josep Mallolas.

## References

[1] Schipper H. Quality of life studies: definitions and conceptual issues. En: Spilker B, ed. Qualityof Life and Pharmacoeconomics in Clinical Trials. 2nd ed. Philadelphia: Lippincott-Raven; 1996:11–23.

[2] Alonso J. La Medida de la Calidad de Vida Relacionada con la Salud en la Investigación y la Práctica Clínica. Gac Sanit. 2000;14:163–7. 10804107 10.1016/s0213-9111(00)71450-6

[3] Porter ME. What is value in health care? New Engl J Med. 2010;363:2477–81. 21142528 10.1056/NEJMp1011024

[4] Haraldstad K Wahl A Andenæs R. A systematic review of quality of life research in medicine and health sciences. Qual Life Res. 2019;28:2641–50. 31187410 10.1007/s11136-019-02214-9 PMC6761255

[5] Black N. Patient reported outcome measures could help transform healthcare. BMJ. 2013;346:f1671–f167. 23358487 10.1136/bmj.f167

[6] Doyle C Lennox L Bell D. A systematic review of evidence on the links between patient experience and clinical safety and effectiveness. BMJ Open. 2013;3:e001570. 10.1136/bmjopen-2012-001570 PMC3549241 23293244

[7] Samji H Cescon A Hogg RS. Closing the gap: increases in life expectancy among treated HIV-positive individuals in the United States and Canada. PLoS One. 2013;8:e81355. 24367482 10.1371/journal.pone.0081355 PMC3867319

[8] Danforth K Granich R Wiedeman D. Global mortality and morbidity of HIV/AIDS. In: Holmes KK Bertozzi S Bloom BR Jha P, eds. MID. 3rd ed. W (DC): TIB for R and D/ TWB 2017 N 3. C 2. P 30212096. No Title, n.d. 30212096

[9] Wandeler G Johnson LF Egger M. Trends in life expectancy of HIV-positive adults on antiretroviral therapy across the globe: comparisons with general population. Curr Opin HIV AIDS. 2016;11:492–500. 27254748 10.1097/COH.0000000000000298 PMC5055447

[10] Deeks SG Lewin SR Havlir DV. The end of AIDS: HIV infection as a chronic disease. Lancet. 2013;382:1525–33. 24152939 10.1016/S0140-6736(13)61809-7 PMC4058441

[11] 90-90-90 An Ambitious Treatment Target to Help End the AIDS Epidemic. Available at: https://files.unaids.org/en/media/unaids/contentassets/documents/unaidspublication/2014/90-90-90_en.pdf [access date March 26, 2023].

[12] Lazarus JV Safreed-Harmon K Barton SE. Beyond viral suppression of HIV - the new quality of life frontier. BMC Med. 2016;14:94. 27334606 10.1186/s12916-016-0640-4 PMC4916540

[13] Akinosoglou K Antonopoulou S Katsarolis I. Patient-reported outcomes in HIV clinical trials evaluating antiretroviral treatment: a systematic review. AIDS Care. 2021;33:1118–26. 33267620 10.1080/09540121.2020.1852160

[14] Bristowe K Murtagh FEM Clift P. The development and cognitive testing of the positive outcomes HIV PROM: a brief novel patient-reported outcome measure for adults living with HIV. Health Qual Life Outcomes. 2020;18:214. 32631444 10.1186/s12955-020-01462-5 PMC7336444

[15] Bristowe K Clift P James R. Towards person-centred care for people living with HIV: what core outcomes matter, and how might we assess them? A cross-national multi-centre qualitative study with key stakeholders. HIV Med. 2019;20:542–54. 31162817 10.1111/hiv.12758

[16] Lazarus JV Safreed-Harmon K Kamarulzaman A. Consensus statement on the role of health systems in advancing the long-term wellbeing of people living with HIV. Nat Commun. 2021;12:4450. 34272399 10.1038/s41467-021-24673-w PMC8285468

[17] Kall M Fresán U Guy D. Quality of life in people living with HIV in Romania and Spain. BMC Infect Dis. 2021;21(Suppl 2):898. 34517820 10.1186/s12879-021-06567-w PMC8436864

[18] Burgui C Guy D Fresán U. Patient satisfaction with HIV care service in Spain: results from a cross-sectional patient survey. AIDS Care. 2022:1–7. 10.1080/09540121.2022.2029818 35102807

[19] Marrone G Mellgren A Eriksson LE. High concordance between selfreported adherence, treatment outcome and satisfaction with care using a nine-item health questionnaire in infcareHIV. PLoS One. 2016;11:e01569161–12. 10.1371/journal.pone.0156916 PMC4911158 27310201

[20] Von Wichmann MA Locutura J Blanco JR. Indicadores de calidad asistencial de GESIDA para la atención de personas infectadaspor el VIH/sida. Enferm Infecc Microbiol Clin. 2010;28:6–88. 10.1016/S0213-005X(10)70048-3 22008585

[21] Oldham J. Integrated care. J Psychiatr Pract. 2013;19:343. 24042240 10.1097/01.pra.0000435033.37685.00

[22] Escarrabill J Almazán C Barrionuevo-Rosas L. Elements clau que influeixen en l’experiència del pacient: patients reported experience measurements (PREM). Barcelona: Agència de Qualitat i Avaluació Sanitàries de Catalunya; 2020. Available at: http://hdl.handle.net/11351/5048.

[23] Reichheld FF. The one number you need to grow. Harv Bus Rev. 2003;81:46–54, 124. 14712543

[24] Jimenez A de Hollanda A Palou E. Psychosocial, lifestyle, and body weight impact of COVID-19-related lockdown in a sample of participants with current or past history of obesity in Spain. Obes Surg. 2021;31:2115–24. 33486709 10.1007/s11695-021-05225-z PMC7826154

[25] Informe “Barómetro de la Sanidad Privada 2020”. Available at: https://www.fundacionidis.com/sala-prensa/notas-de-prensa/informe-barometro-de-la-sanidad-privada-2020 [access date March 26, 2023].

[26] Raventós S. El Net Promoter Score es la forma más eficiente de medir la experiencia del paciente. Available at: http://sanidadprivada.publicacionmedica.com/noticia/el-net-promoter-score-es-la-forma-mas-eficiente-de-medir-la-experiencia-del-paciente [access date March 26, 2023].

[27] Coulter A Locock L Ziebland S. Collecting data on patient experience is not enough: they must be used to improve care. BMJ. 2014;348:g22251–g2225. 10.1136/bmj.g2225 24671966

